# Zika virus public health crisis and the perpetuation of gender inequality in Brazil

**DOI:** 10.1186/s12978-021-01067-1

**Published:** 2021-02-15

**Authors:** Raquel Zanatta Coutinho, Aida Villanueva, Abigail Weitzman, Letícia Junqueira Marteleto

**Affiliations:** 1 Faculdade de Ciências Econômicas, Universidade Federal de Minas Gerais and Center for Development and Regional Planning (Cedeplar), Av. Pres. Antônio Carlos, 6627-Pampulha, Belo Horizonte, MG 31270-901 Brazil; 2 Department of Sociology, University of Massachusetts-Amherst, 910 Thompson Hall, Amherst, MA 01003 USA; 3 The College of Liberal Arts, University of Texas at Austin and Population Research Center, 116 Inner Campus Dr Stop G6000, Austin, TX 78712 USA

**Keywords:** Zika, Public health campaigns, Gender norms, Gendered power within couples, Negotiations of sex and contraception, Contraception management, Unplanned pregnancy

## Abstract

**Background:**

In 2015–2017, the Americas experienced a highly consequential epidemics for pregnancy and childbearing. Mainly transmitted by the mosquito *Aedes aegypti*, but also through sexual intercourse, the Zika virus poses the risk of congenital Zika syndrome to fetus, which includes microcephaly and other child development complications. When a public health crisis taps directly into reproductive health, typically a feminine realm, responses to the emergency may exacerbate deeply-rooted gender norms. This paper investigates the role of gender in two relational contexts: (a) the government-led response to the pandemic in terms of communication campaigns aimed at preventing Zika infections; and (b) an individual level of response to the emergency, concerning women’s negotiation with their sexual partners with regard to the prevention of Zika as well as pregnancies.

**Methods:**

We conducted content analysis of 94 unique pieces from public health communication campaigns produced by governmental agencies with the goal of promoting Zika awareness. Print and online materials were collected from May 2016 to August 2017, and included TV ads, Internet Pop-ups, and pamphlets. We also analyzed transcripts from 16 focus groups conducted with reproductive-aged women (18–40) in Belo Horizonte and Recife, two large cities differently affected by the Zika outbreak. Women answered open-ended questions connected to the epidemic, in areas such as personal knowledge and experiences with the Zika virus, experiences of their friends and acquaintances, their primary information sources, their perceptions of public health efforts toward containing the outbreak, as well as women’s contraceptive use.

**Results:**

Campaign pieces handling pregnancy and microcephaly were largely gendered. Pieces targeted women, placing on their shoulders the responsibility for protecting a potential fetus from the disease. Importantly, campaigns neglected addressing male’s participation on Zika prevention and contraceptive management, while failing to take into account Brazil’s large proportion of unplanned pregnancies. Women were placed in a double bind by being expected to prevent both pregnancy and Zika, in a context where gendered power imbalances often translate in women having little power/means for condom negotiation/avoiding unprotected sexual intercourse.

**Conclusion:**

Government and individual responses to the epidemics reinforced gender roles, situating pregnant women as responsible for averting mosquito bites and microcephaly. Further, prevention campaigns largely excluded men. Since low-socioeconomic status women possessed fewer resources to preclude infection, we also found that beyond the gender divide, this subgroup faced more pronounced Zika prevention challenges as they found it harder to negotiate condom use with their sexual partners and often could not access other types of contraceptives resulting in unplanned pregnancies.

## Plain English summary

This paper investigated the role of gender in two relational contexts: public health messaging and women’s Zika and pregnancy prevention negotiation with their partners during a public health shock of international proportions, the Zika outbreak in Brazil. Combining content analysis of public health campaigns with unique focus group data collected amidst the epidemic in two large cities in Brazil, we find that the government and individual responses to the epidemics reinforced gender roles, emphasizing prevention of both disease and vector among women while excluding men. Traditional gender roles placed women in a double bind by expecting them to prevent pregnancy and Zika without challenging the normatively gendered power dynamics hindering them from doing so. Public health campaigns perpetuated female vulnerability to infectious diseases and unplanned pregnancy, especially among low socioeconomic status women. Women’s understanding of their role in prevention was strongly linked to gender ideology and magnified disadvantages among women of low socioeconomic status.

## Background

In 2015–2017, the Americas experienced a highly consequential epidemic for pregnancy and childbearing. Mainly transmitted by the mosquito *Aedes aegypti*, but also through sexual intercourse, the Zika virus poses the risk of congenital Zika syndrome to the fetus, which includes microcephaly and other child development complications [1]. In November 2015, when the link between the Zika virus infection and the surge in cases of microcephaly was established, the government of Brazil—the country most affected by the outbreak—issued a public emergency announcement [2]. The announcement sparked mass media and government campaigns, revealing the potential risks to pregnant women and their babies. Images of microcephalic babies gained the headlines around the world. Brazil’s Health Ministry informally recommended that women should avoid pregnancy until the risk had subsided [3–5], and in April 2016 the World Health Organization (WHO) declared Zika a Public Health Emergency of International Concern [6].

By November 2020, 3563 cases of microcephaly were confirmed in Brazil [7], disproportionately affecting poor, black, rural women [8, 9]. The Zika epidemic impacted birth rates and fertility [10], and fertility rates declined more steeply among young and highly educated women, which means that the informal recommendation to delay pregnancy was not followed by the group most affected by microcephaly [11].

As gender scholars have pointed out, structural socioeconomic and gender inequalities are likely to exacerbate the consequences of the epidemic. For example, recommendations to delay pregnancy assume all women are able to implement their reproductive plans. However, preventing pregnancy in Brazil is a highly complex matter [4, 5], as 55% of pregnancies carried to term are unintended [12]. There are several barriers to contraceptive implementation, such as lack of access and high costs [13, 14], inconsistent use [15], persistent reliance on methods with high failure rates, and overall lack of access to medical care [16]. Further, gender-based power dynamics, including intimate partner violence, often translate to women having little ability to negotiate condom use and other behaviors that could prevent both pregnancy and sexual transmission of Zika [17–23]. Importantly, all these vulnerabilities are more prevalent among low-SES, less educated women, compared to their more advantaged peers [18, 24–26]. Women who lack reproductive rights are among the poorest and least educated in the country, and oftentimes, live in the least developed areas of the country. They also lack access to clean water and sanitation, which exposes them even more to the risks of getting infected by mosquito borne diseases. This fatal combination explains why the impact of the Zika virus fell most heavily on the most disadvantaged members of society [5, 8, 9, 16, 27–35].

While highly relevant, most of the literature regarding the gendered consequences of the Zika outbreak has been theoretical [5, 16, 27–35], with a dearth of empirical studies exploring how traditional gender stereotypes increase women's vulnerability to Zika contraction and to how gendered divisions of labor hinder arbovirus control. The few exceptions to this are studies that have explored contraceptive use and fertility intentions and behavior [24, 28, 36].

When a public health crisis taps directly into reproductive health, typically a feminine realm, gender systems may reproduce traditionally and deeply rooted gender norms, especially those linked to cultural beliefs and expectations playing out in different relational contexts. This study investigates gender norms in *government-level* and *individual-level* contexts to prevent pregnancy and Zika infection amidst the first year and a half of the epidemic. We examined three research questions: Did public health campaigns reinforce heteronormative gender norms? What role did gender play in shaping how women navigated Zika and pregnancy prevention during the epidemic with their partners? And, did social class differences significantly affect how women negotiated the threat of Zika infection?

### Gender conventions, Zika and pregnancy prevention at the government and individual levels

In late 2015, as the country declared a state of emergency and anxiously confronted the Zika epidemic [3], headlines and media coverage highlighting the upsurges in microcephaly framed women’s protagonism in the war against the mosquito [29, 30, 35, 37]. Besides the media, two primary relational contexts modeled gendered Zika prevention perceptions. The first was at the government level, through public health communication campaigns seeking to raise awareness about the new virus. The second was at an individual level, through intimate unions, since Zika can be transmitted through sexual intercourse and exposure to mosquito bites. As known, the latter is the site of family labor, including household chores needed to prevent mosquitos from breeding*.*

#### Government level

According to Charaudeau [38], public health campaigns differ from advertising campaigns because the audience does not represent a consumer, but rather, a person with a civic and moral duty to modify behavior in the name of social solidarity. Brazilian public health campaigns historically have centered on two major diseases, HIV/AIDS and dengue. Both campaigns are informative for understanding the Brazilian communication strategy targeted at containing the Zika epidemic. While HIV is sexually transmitted, dengue is acquired only through the bite of the *Aedes aegypti* mosquito. Zika shares both transmitting vectors.

In the case of HIV, the Brazilian public health sector’s response has been widely celebrated as successful in altering behavior [39–41]. In addition to a universal treatment policy and large-scale condom distribution, national TV and print campaigns promoted condom use, seeking prompt treatment, as well as fighting stigma. Scholars described such campaigns as liberal, praising the use of an open language regarding sexual intercourse. While successfully containing the disease among high-risk populations [41], such as gay man and sex workers [42], this strategy unintentionally alienated other groups; namely, adult women who were primarily infected through sexual relations in monogamous, stable relationships [18, 43, 44]. These women encountered challenges to enforcing condom use with their (purportedly) monogamous partners [18, 19, 21, 22]. In fact, married women displayed the lowest proportion of condom use compared to other demographic groups [45].

In contrast, dengue remains a pervasive public health problem in Brazil, with public health efforts starting decades ago. In the light of poor results, a new wave of massive public health campaigns started in the 1990s, aimed at educating Brazilians on how to prevent the breeding of its carrier, the *Aedes* mosquito [46]. Widely disseminated materials outlined measures to eliminate mosquito-breeding sites, primarily instructing on the removal of stagnant water sources and managing solid waste in households and neighborhoods [47, 48]. Yet, these efforts were unsuccessful at reducing either the incidence of dengue or the proportion of severe cases [29, 46].

Further, dengue campaigns were drastically gendered. Typically, campaign pieces presented women watering plants and doing household chores while men performed ‘outdoor chores,’ like cleaning rain gutters. The gendered nature of the campaigns is an important component for understanding the persistence of dengue over the years [49, 50] and have also been explored linked to other health crisis [16, 51].

In the midst of the national Zika threat and microcephaly, public health and governmental offices around the country launched several campaigns to inform the public on how to eradicate the mosquito, unsurprisingly resembling the dengue-eradication initiative. In addition, Zika campaigns informed the public that, if pregnant, women should implement measures of protection to avoid mosquito bites, such as using repellants or long sleeve clothing, in order to prevent the risk of microcephaly.

Scholars have argued that the images of microcephaly, widely broadcasted during the course of the epidemics, may have overstated the gendered nature of the epidemic [5], causing an emotive response [52] followed by an immediate feeling of empathy, but one that detaches maternity from women´s health [30]. As Davies and Bennett [16] point out, “within this narrow framing, women are seen as either caregivers or mothers when it comes to healthcare access and rights” (pages 1042–1043).

Given this historical, epidemiological, and policy context, we expect that the Brazilian public health campaign to prevent Zika primarily charged pregnant women with this responsibility [35]. This is problematic not only from a gender justice perspective, but also from a focus on controlling the pandemic. For instance, a woman may follow all protocols related to mosquito bites, and become infected by intercourse with a partner who does not use condoms. That is, men’s behavior is important for preventing Zika and excluding them from the campaign is likely to be consequential. Relatedly, a focus on pregnant women also leaves non-pregnant women susceptible to the Zika virus by minimizing the message that they too should actively take precautions. For example, in Borges et al.’s study [36], with a large representative sample in an area in the Brazilian Northeast, only 50.2% of the women interviewed knew Zika could be sexually transmitted or had heard the recommendation to use condoms to prevent Zika transmission. Only 1 in 10 women were asked about their pregnancy intentions by their health care provider and only 14.4% were advised about condom use to prevent Zika infection. In a study conducted in the Northeast Brazil, Diniz and colleagues found that none of the young women interviewed knew about the sexual transmission of Zika [28]. The latter point is particularly concerning given the high rates of unintended pregnancy in the country, especially at young ages.

#### Individual-level

When it comes to interactions at the individual-level, it is necessary to address women’s relationships with their intimate partners. Relationships with intimate partners are key to women’s behaviors connected to the prevention of sexual transmitted infections (STIs) and unplanned pregnancies, as well as household tasks related to health behavior. As noted, condom practice and pregnancy prevention are of primary interest when analyzing the Zika epidemic, giving the sexual transmission and the risk of microcephaly. Importantly, unwanted pregnancies increase chances of fetal microcephaly, as women might take longer to test for pregnancy, have less willingness or incentive for adopting healthy behaviors [53, 54] or delay the starting point at which they begin protecting themselves against mosquito bites [24].

Existing literature on contraceptive practices shows how passivity, submission, and emotion illustrates the context in which many sexual relationships occur in Brazil, and since protection is not always discussed in advance, women sometimes have to ask their partner to use condom during intercourse, oftentimes resulting in unprotected sex [17, 21–23]. Brazilian women who “would not even try to stop sexual intercourse to ask the partner to put on condoms” or “would not stop the sexual intercourse in case they changed their minds about having sex” are almost four times more likely not to use condoms compared to women who report being able to pause or stop intercourse [22].

Giving that campaigns may have failed to address these contexts of gender inequality [5, 30, 34] and women´s limited ability to implement their reproductive plans [27, 29, 32], we expect that the Zika epidemics did not change the way couples make sexual and reproductive decisions.

Women’s socioeconomic status is closely tied to their ability to avoid sexual behaviors associated with the risk of STI transmission. Ethnographic research has also illuminated how poverty leaves low-SES women vulnerable to intimate partner violence and impedes their sexual agency including ability to make decisions about condom use [23]. In Brazil, higher educational attainment was associated with lower adolescent and unintended pregnancy [15, 55], and more confidence in enforcing condom use [25], resulting in higher condom practice [26, 45].

It is also important to consider that existing research shows hegemonic gender views remain more prevalent among the working-class [56, 57] as a number of contributions have addressed this topic in connection to the gender distribution of household labor. Low-SES women assume men are incompetent for housework, while, in a broader sense, low-SES women encounter more obstacles for exerting relationship power [58]. In contrast, middle-class women exercise more egalitarian relationships with their partners and therefore accomplish a more balanced domestic duty distribution.

We therefore expect a drastic class variation in Brazilian women’s bargaining power during the Zika outbreak, with low-SES women showing less power to negotiate an egalitarian distribution of responsibilities pertaining to Zika prevention compared to their high-SES peers.

## Methods

In order to address our research questions, we draw upon two data sources—content analysis of public health campaigns and focus groups. Further, as outlined above, we use two levels of analysis, government and individual.

First, to answer the first question, *Did public health campaigns reinforce heteronormative gender norms?,* we conducted a content analysis of 94 unique campaign pieces, mass-produced by governmental agencies [59–61]. These pieces are aimed at promoting Zika awareness and had a broad targeted audience, from diverse sociodemographic backgrounds and lifestyles. We chose two specific city capitals, Belo Horizonte (state of Minas Gerais) and Recife (state of Pernambuco), in addition to the federal government as governmental agencies producers of campaign pieces. The two city municipalities were chosen due to their varying infrastructural development and Zika incidence, as the former is in the more developed southern state of Minas Gerais, while the latter is in the less developed northeastern state of Pernambuco, the region where Zika first started and with the highest number of cases of microcephaly in the first year of the epidemics.

Materials were collected from May 2016 to August 2017, and entailed material publicly available for download, directly or upon request, and dissemination during the targeted Zika outbreak period.^1^

Coding built on existing dengue public health campaigns [49], gendered Brazilian TV ads [50] and gender advertising [59]. Two research team members scrutinized and coded pieces side-by-side, following deductively and inductively-derived categories [62]. Eventually, when an unanticipated code emerged, the new coded was added, and the previous pieces were re-examined.

All campaign pieces were coded with information on the governmental institutions that produced them, the channels through which they were broadcasted (e.g. TV ad, radio ad, radio jingle, internet posts or pop-ups, print posts, billboards, busdoors, wall banners, folders, booklets and infographic), the perceived audience for which they were produced (general population, person responsible for the household, women, men), the more preponderant color scheme (pastel and soft colors, red, yellow and bright, other colors), the main enunciator (the person or institution whose voice is asking for a change of behavior or habits), presence of graphic elements (e.g. mosquito with the bar or governmental logo), their main topic or message (e.g. information about symptoms of Zika, information about getting rid of standing water, information about sexual transmission and importance of condom use, information on measures of self-protection regarding mosquito bites such as use of repellent or long sleeved clothing, information about microcephaly, information about importance of seeking medical care, among others), the characters illustrated in the material (e.g. pregnant women, women, men, mosquitos, children, baby), and whether microcephaly was referenced. For TV material, we also coded relevant elements such as the physical appearance of the character (age, clothing style, race and ethnicity, gesture, position and location), scenario and social class (type of household, conditions of the street), message/actions/activities performed by characters, atmosphere/impression (happy, scared, romantic), among others.

To further address the first research question and to answer the second and third research questions, *What role did gender play in shaping how women navigated Zika and pregnancy prevention during the epidemic with their partners?* and, *did social class variations significantly affect how women negotiated the Zika infection threat?,* we examined transcripts from 16 focus groups [24] with reproductive-aged women (ages 18–40) conducted also in Belo Horizonte and Recife.

Within both cities, we identified four large neighborhoods to sample, in order to stratify the groups by socioeconomic status and environmental risk. These neighborhoods were carefully selected based on three criteria: differing proximities to fresh bodies of water, contrasting physical infrastructures (sky rise apartments versus single-story homes), and the presence of segregated but adjacent high and low socioeconomic status residences. The first criteria—differing proximities to fresh bodies of water—should affect the presence of *Aedes aegypti* mosquitoes and potentially women’s beliefs about their risk of transmission. The second—contrasting infrastructures—should also influence women’s beliefs about their risk of transmission because mosquitos are not able to fly to high elevations. In other words, women residing in sky-rise apartment buildings generate an overall sample that is diverse in terms of race/ethnicity, age, and socioeconomic background, and may feel more protected from the virus than women who reside in one-story homes. Finally, the third criterion—segregated but adjacent populations with differing socioeconomic statuses—should result in the participation of women whose ability to prevent Zika and unintended pregnancies substantially differ.

All participants were recruited from central locations within each neighborhood such as outside grocery and convenience stores. All focus groups followed the same interview protocol (available at the Additional file 1) and were moderated by the first and fourth authors of this paper. Women answered broad, open-ended questions related to personal knowledge of and experiences with the Zika virus, the related experiences of their friends and acquaintances, their primary information sources, and their perceptions of public health efforts and women’s contraceptive use. When necessary, moderators made additional comments to probe women in order to obtain complete answers to a topic. Gender ideology arose organically during most conversations but did not entail an explicit question included in the focus group protocol.

## Results

### Did public health campaigns reinforce heteronormative gender norms?

**Fig. 1:**
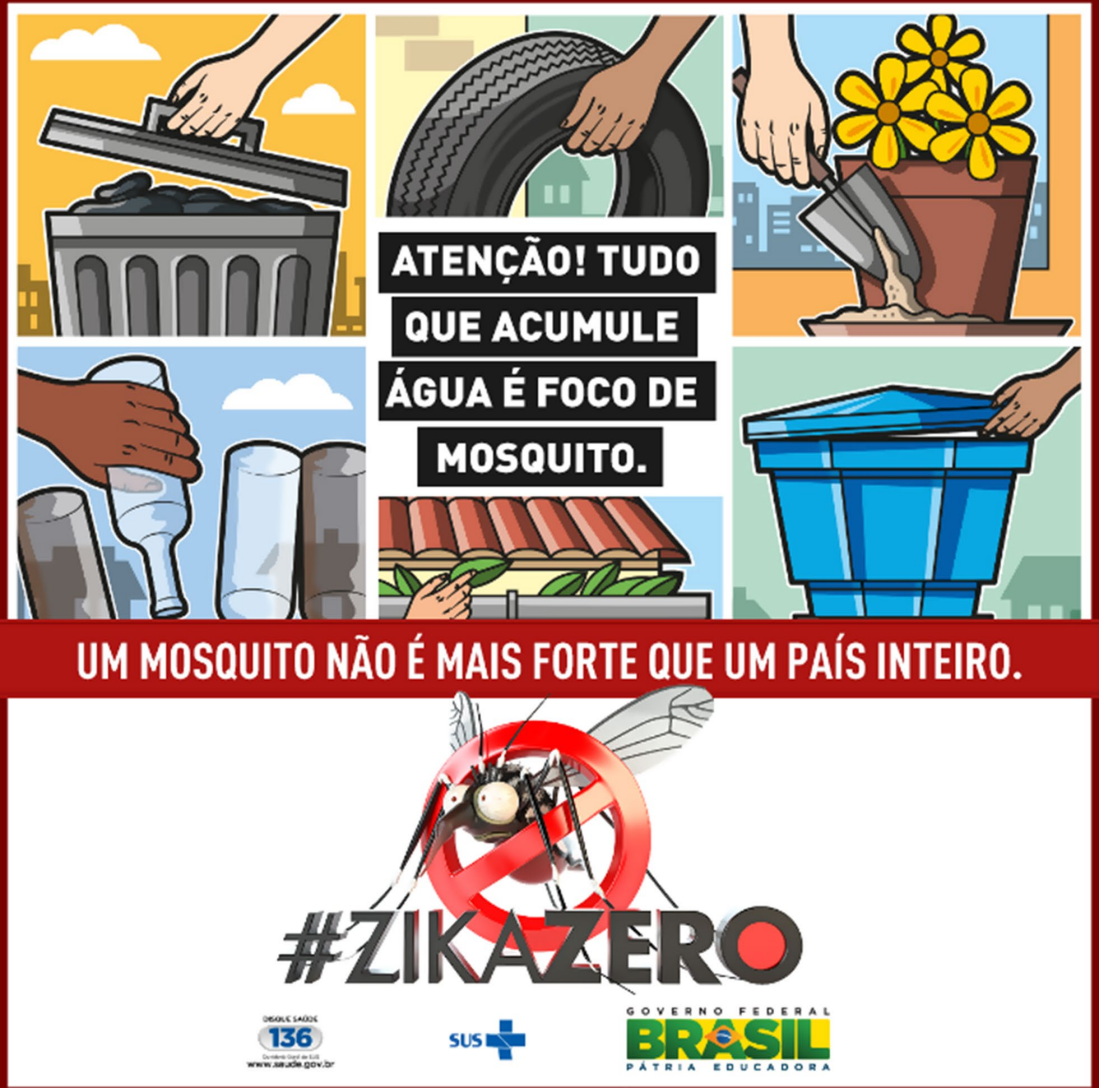
Internet/print media pop up. Translation: *Attention! Everything that accumulates water is a focus for mosquito breeding. One mosquito isn´t stronger than a whole country*

We start by examining the campaign items that Brazilian governmental agencies disseminated, aimed at haltering the Zika virus epidemic. Descriptive statistics are available on Table 1. Out of 94 pieces, about a third (32) were short internet or print posts (Fig. 1), followed by folders (16), posters (14) and TV ads (11).

**Table 1:** Description of type of Zika public health dissemination campaign materials by institution which produced it, main messages and main perceived audience; Brazil, May 2016–August 2017. Source: Communication campaigns pieces downloaded from public resources at each institutional website (Ministério da Saúde, MG State, PE State, Townhouses of Belo Horizonte and Recife)

	Busdoor	Poster	Brochure	Folder	Infographic	Radio jingle	Billboards	Radio ad	TV ad	Post	Total
Panel A											
Institution											
Minas Gerais State (MG)	0	1	0	2	0	0	0	0	0	0	3
Pernambuco State (PE)	0	1	0	1	0	0	0	0	1	0	3
Ministério da Saúde	2	8	2	5	2	5	3	0	9	32	68
Belo Horizonte	0	1	1	4	0	0	0	0	0	0	6
Recife	0	3	0	4	0	0	0	6	1	0	14
Total	2	14	3	16	2	5	3	6	11	32	94
Panel B											
Message^a^											
Eliminate standing water	2	12	3	16	1	5	3	6	11	20	79
Personal protection against bites	0	3	2	7	1	0	0	0	4	0	17
Special care during pregnancy	0	4	2	5	1	0	0	0	4	0	16
Information about Zika Symptoms	0	1	3	10	1	0	0	0	2	4	21
Seek health care	0	5	2	7	0	0	0	0	2	1	17
Pregnancy counseling	0	3	1	2	0	0	0	0	0	0	6
Codom use or contraception	0	0	1	2	0	0	0	0	0	0	3
Microcephaly	0	5	2	3	1	3	0	0	5	3	22
Other^b^	0	1	0	0	0	1	0	0	0	5	7
Total	2	34	16	52	5	9	3	6	28	33	188
Panel C											
Main perceived audience^c^											
General public	1	5	2	3	1	4	1	3	4	24	48
Person responsible for household	1	4	0	9	0	1	2	3	3	8	31
Women	0	5	1	4	1	0	0	0	4	0	15
Total	2	14	3	16	2	5	3	6	11	32	94

^a^Pieces may contain more than one message. So, total count of messages (188) exceeds total count of pieces (94)

^b^Pieces that solely contained statistics, images without any phrase or information about what the government is doing to prevent Zika were classified as Other (7)

^c^Pieces coded according to most relevant perceived audience. As each piece received just one classification, pieces were coded in the following order of relevance: woman, person responsible for household, general public. Thus, if a piece contained information target at both woman and person responsible for household, in this question it was classified as 3 (woman) as this category is more relevant for the analysis

Brazil’s Ministry of Health generated around 70% (68 of 94) of the analyzed material and has produced a more diverse range of materials compared to the other institutions (Table 1, panel A). Although state and municipal administrations also have their own campaign materials, they more often reproduced the materials made available by the Ministry of Health.^2^

Table 1, panel B brings a description of the main topic or messages being delivered by the type of communication strategy. As each communication piece may carry more than one message the total count (188) refers to the number of messages, not the number of pieces.

The same communication strategies against dengue and the mosquito *Aedes aegypti* were used amidst the Zika emergency. Seventy-nine of 94 pieces (almost 85%) contained information on how to destroy mosquito breeding sites by not allowing standing water to occur (Table 1, panel B). A mosquito is present in 68 out of the 94 pieces, either as a picture or drawing (not shown). The colors used in these pieces usually remained gender-neutral, typically bright yellow and red conveying urgency (percentage not shown).

The fact that Zika can be transmitted from a pregnant woman to her fetus, causing microcephaly, is a vital distinction between the Zika threat and dengue. Nevertheless, much fewer are the pieces containing information on microcephaly (22), Zika symptoms (21), personal protection measures against mosquito bites and information about importance of seeking health care (17 each) and taking additional care during pregnancy (16) (Table 1, panel B). Only 6 pieces mention the necessity of speaking to a physician if one wishes to get pregnant and only 3 out of 94 pieces mentions the necessity of wearing condom or contraception during the epidemics. Not a single TV or Radio ad, or even short post mention the necessity of using condom or contraceptive or the importance of talking to a health professional in case one wishes to get pregnant during the Zika epidemics. Importantly, none of the pieces, not even the ones about contraceptive use provides information about sexual and reproductive health (SRH) services free of charge in public health clinics. Besides, none addresses contraceptive negotiation in intimate partner union.

In order to evaluate the main perceived audience (Table 1, panel C), each piece was recoded in order to assign a single category of main perceived audience. Further, we applied the following order of importance: woman, person responsible for household, general public. Thus, if a piece contained information targeted at both woman and/or a person responsible for the household, the category was classified as “woman” as this category is more relevant for the analysis. None of the 94 pieces were targeted to men. Taken as a whole, campaign pieces using a single phrase to promote mosquito eradication (posts, billboards and busdoors) did not target one specific gender, as the proportion of pieces targeting the general population and sometimes the person responsible for the household were much higher (Table 1, panel C). However, posters, folders and TV ads seem to aggregate more messages towards women.

On Table 2, the 188 Zika public health dissemination campaign materials´ main messages were then analyzed according to their main perceived audience and presence of female and male figures on the piece. The 79 messages regarding eliminating standing water, a recommendation categorized as housework, almost equally targeted the general population (35) and the person responsible for the household (31) without having a strict gendered responsibility expressed. Nevertheless, 13 of those 79 pieces depicted women as a perceived audience (none depicted men). Regarding messages about Zika symptoms: out of 21 messages, 12 were targeted at the general public, 4 the responsible for the household and 5 to women.

**Table 2:** Description of Zika public health dissemination campaign materials’ main messages by main perceived audience and existence of female figure and male figure; Brazil, May 2016–August 2017. *N* = 188. Source: Communication campaigns pieces downloaded from public resources at each institutional website (Ministério da Saúde, MG State, PE State, Townhouses of Belo Horizonte and Recife)

Main audience*	Eliminate standing water	Seek health care	Special care during pregnancy	Pregnancy counseling	Condom use or contraception	Microcephaly	Information about Zika symptoms	Personal protection against bites	Other
General public	35	6	3	0	0	9	12	3	7
Person responsible for household	31	2	0	0	1	2	4	2	0
Women	13	9	13	6	2	11	5	12	0
Female figure (woman or pregnant)									
No	51	7	4	1	1	9	13	4	7
Yes	28	10	12	5	2	13	8	13	0
Male figure									
No	55	10	10	5	1	14	15	10	0
Yes	24	7	6	1	2	8	6	7	1
Total	79	17	16	6	3	22	21	17	7

^a^Pieces coded according to most relevant perceived audience

But when it comes to messages about seeking health care, taking special care during pregnancy, talking to physician if wishes to get pregnant, condom use or contraception, information about microcephaly or measures of personal protection against bites, the messages are heavily gendered as women (especially pregnant) are the main perceived audience of those messages and were also more often illustrated at the pieces by the utilization of images, drawing or narrating voices (Table 2). Out of 22 total items tackling microcephaly, 11 targeted women directly. Women were portrayed in 13 of those pieces, being depicted by themselves in five of them. In the 17 pieces about personal protection about mosquito bites (last column on Table 2), women are depicted 13 times, almost double the times that males were depicted (7). Babies, microcephalic or not, appear in 7 pieces (not shown).

Since some announcements were more informational or educational about the disease, they sometimes encompassed lengthy text. However, others directed the audience using a single phrase (“Get rid of standing water in your backyard”) without telling them *why* to do it. Table 1 at the Additional file 2 brings an analysis of 72 pieces that were in print format (excluding TV and Radio of any sort) comparing the 21 pieces that included lengthy texts conveying more information (brochure, folder and infographic) with the 51 remaining pieces which contain short messages. Lengthy materials require more effort from the reader to extract information. In the lengthy ones, women continue to be portrayed more often than men and the concern with Zika symptoms (14 out of 21) and the information about the measures of protection against mosquito bites (10 out of 21) receive substantial attention.

#### Lengthier pieces

We now dive into campaigns where the scenario of gender becomes even more evident. First, we will discuss the 21 print media with long texts, and then we will analyze some examples that include TV ad.

For print media with long texts only (comprised of brochure, folder and infographic), Table 3 shows the proportion of pieces in which females and males are depicted and also the main perceived audience for campaigns handling personal protection against bites (Table 3, panel A), microcephaly (Table 3, panel B), and special care during pregnancy (Table 3, panel C).

**Table 3:** Description of Zika public health dissemination campaign by subjective (microcephaly, measures of personal care to prevent mosquito bites), main perceived audience and presence or male and female figures for only print pieces with long length (*N* = 21); Brazil, May 2016–August 2017. Source: Communication campaigns pieces downloaded from public resources at each institutional website (Ministério da Saúde, MG State, PE State, Townhouses of Belo Horizonte and Recife)

	Female		Male		Main perceived audience		
	Absent	Present	Absent	Present	General public	Person responsible for household	Women
Panel A							
Not about personal protection against bites	10	1	10	1	3	8	0
Personal protection against bites	4	6	7	3	3	1	6
Panel B							
Not about microcephaly	12	3	13	2	4	9	2
About microcephaly	2	4	4	2	2	0	4
Panel C							
Not about special care during pregnancy	11	2	11	2	41	22	2
Special care during pregnancy	3	5	6	2	1	0	7

Long text materials comprised of Brochure, Folder and Infographic

Women are the stated audience in 60% (6 out of 10) of pieces about personal protection about bites. When it comes to pieces containing microcephaly in its subject (6 pieces in total), the presence of females is more pronounced than in comparison with males, who tend to be more absent from those pieces (Table 3, panel B). Four out of 6 long-length pieces about microcephaly were perceived as being intended for females (66%).

For pregnancy, the story is even more striking and those pieces drastically target women, placing the responsibility for protecting a potential fetus from the disease on females. Seven out of 8 pieces (85%) only targeted women (Table 3, panel C). Besides content, these pieces typically used pastel colors, a decision further communicating the perceived audience: pregnant women (not shown).

**Fig. 2:**
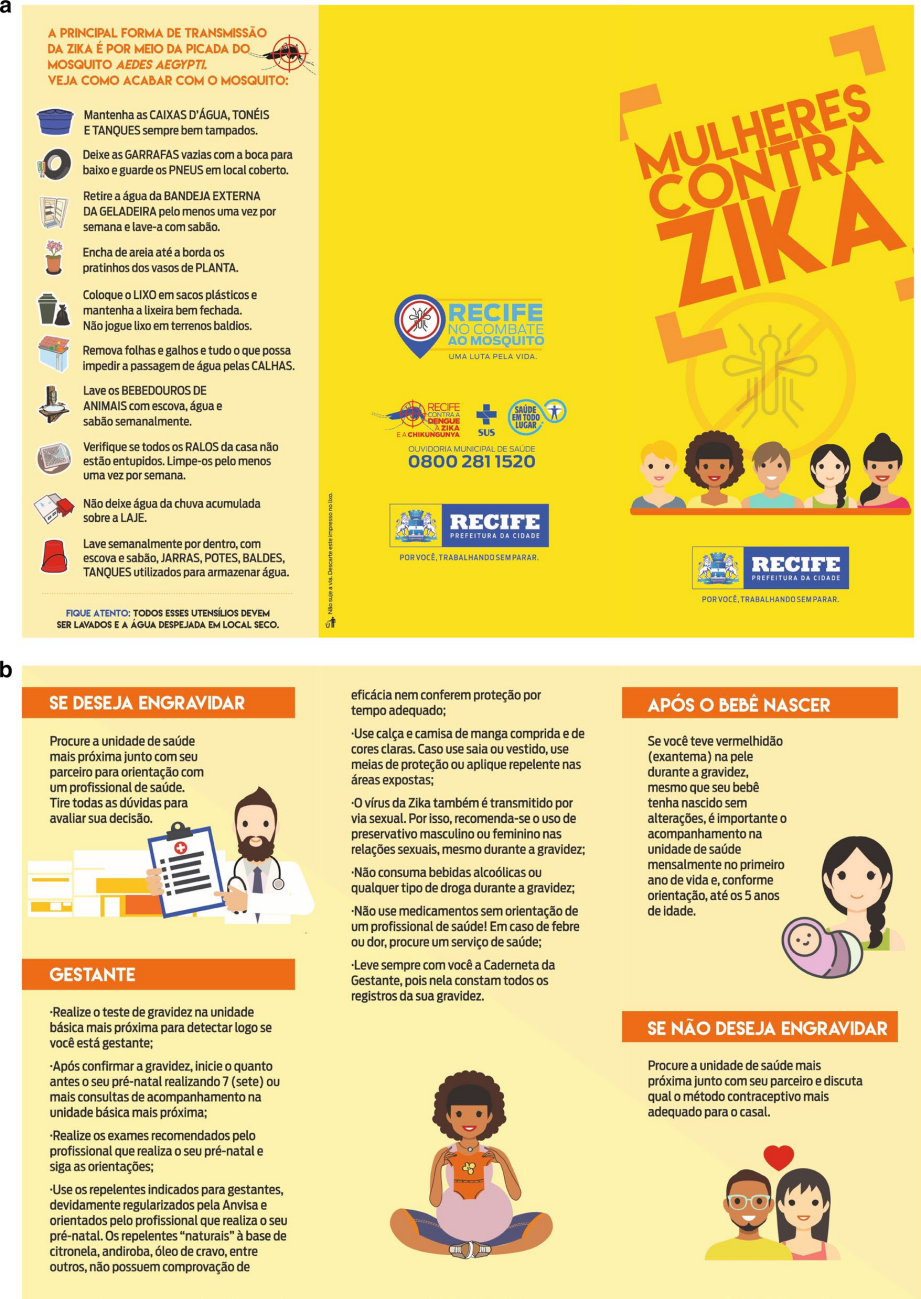
**a** Front side of the folder prepared by the city of Recife; **b** Backside of the folder prepared by the city of Recife

**Fig. 3:**
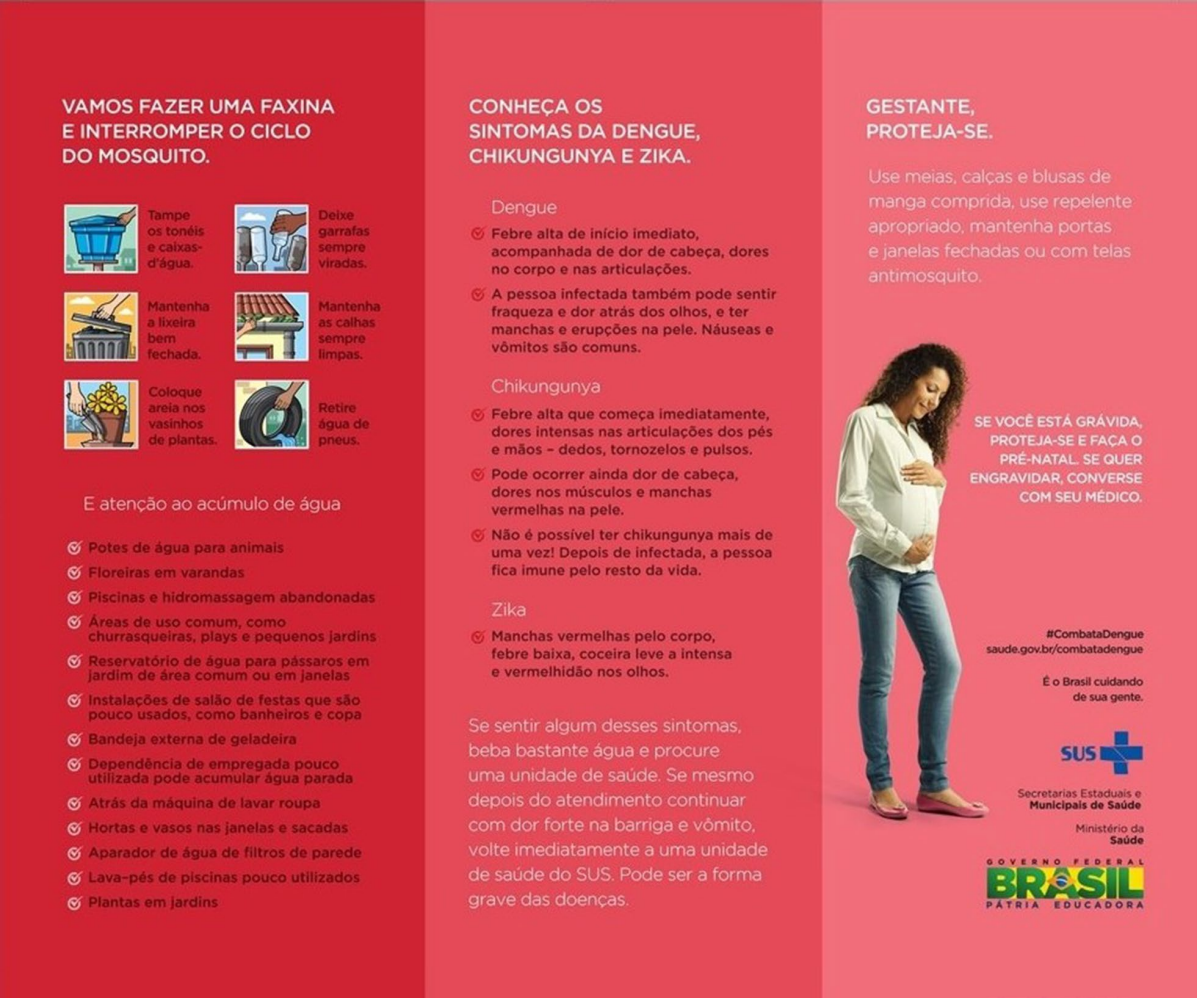
Folder prepared by the Ministry of Health

**Fig. 4:**
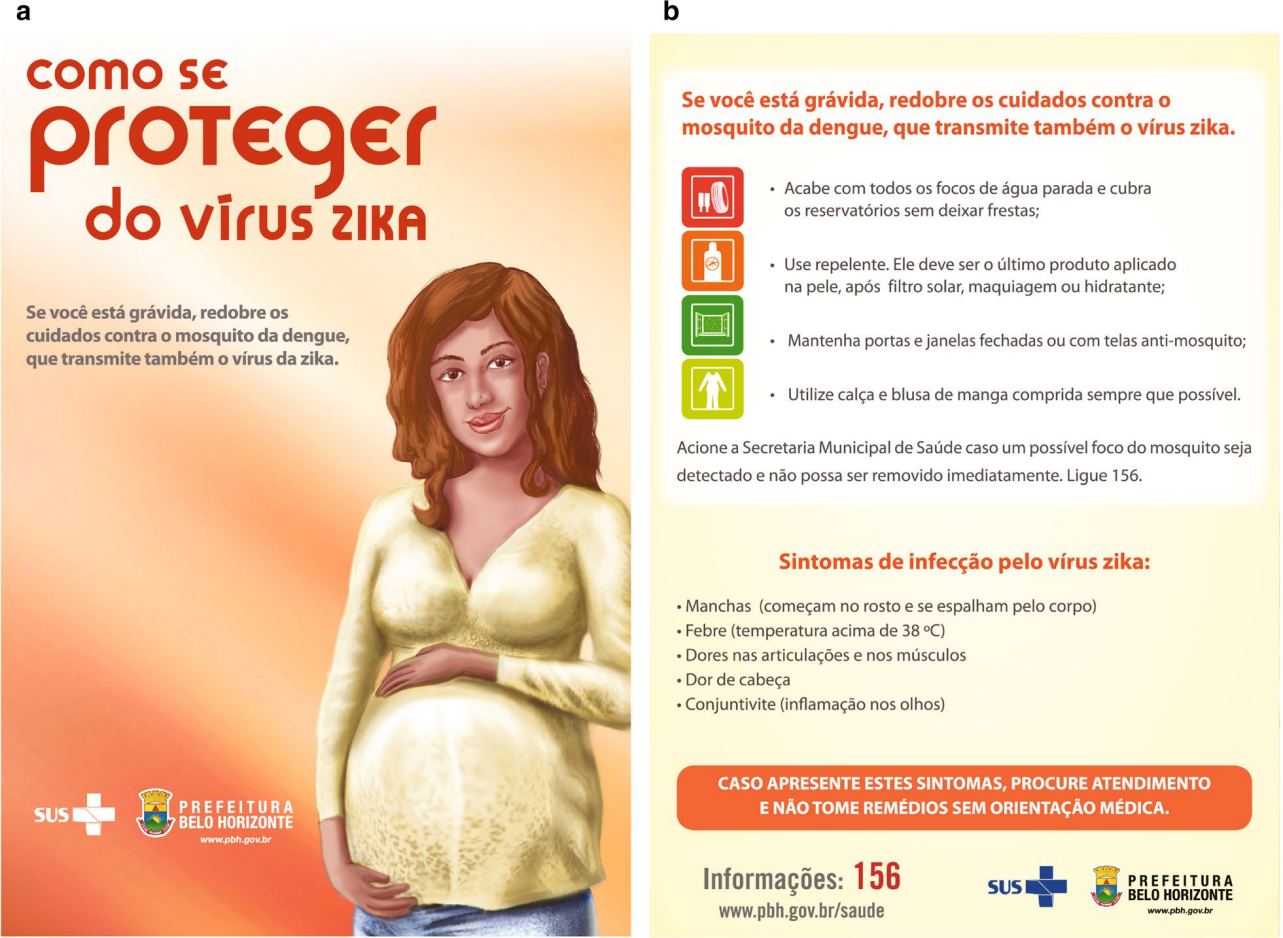
**a** Front side of the folder prepared by the Municipal Government in Belo Horizonte; **b** Backside of the folder prepared by the Municipal Government in Belo Horizonte

We next present some examples of communication pieces. Figure 2a presents one pamphlet with the headline “Women against Zika.” Other typical headlines read “Pregnant Lady: Protect Yourself” (Fig. 3) and “If you are pregnant, protect yourself and go to prenatal care. If you want to get pregnant, talk to your doctor”. Using female pronouns exclusively, the announcements directly referenced women (see Figs. 2b and 4b) and intensively focus on pregnancy [5, 35].

The pamphlets displayed in Figs. 3 and 4a portray pregnant women with hands placed on their bellies.

One Brazilian federal campaign TV ad (see pictures in Additional file 3, Fig. 1a, c) displayed the protagonist—a young pregnant woman—walking around her home explaining the fetal microcephaly risk and how to prevent mosquito breeding within the dwelling, as well as mosquito bites. Her husband carried a bucket in the background. The piece concludes with the protagonist sitting with two male family members, likely her partner and adolescent son, watching TV. Only she (the protagonist) talked to and engaged with the audience, addressing women exclusively. Therefore, the piece clearly charged women with Zika containment and presented females as family health prevention *experts* while men remained disengaged, even as prevention *subjects*.

We uncovered further pieces explicitly assigning women the familial protection responsibility [16, 30]. Thus, the flier portrayed in Fig. 4a, b openly advised a *pregnant woman* to protect her dwelling against the mosquito. These campaigns situated women as *competently* dealing with pregnancy and caring during the Zika epidemics while the male remained detached from caring and parenting responsibilities [63]. Across all materials we examined, we did not find a single piece *speaking directly to* men.

Males were often depicted in as, as can be seen on Table 2, but were rarely the protagonists, sometimes just lending parts of their bodies or assuming a secondary role. In many cases, they lent their voices to narrate a radio ad or posed on print materials as a physician, both roles that imply a position of power, not of victimization or responsibility.

**Fig. 5:**
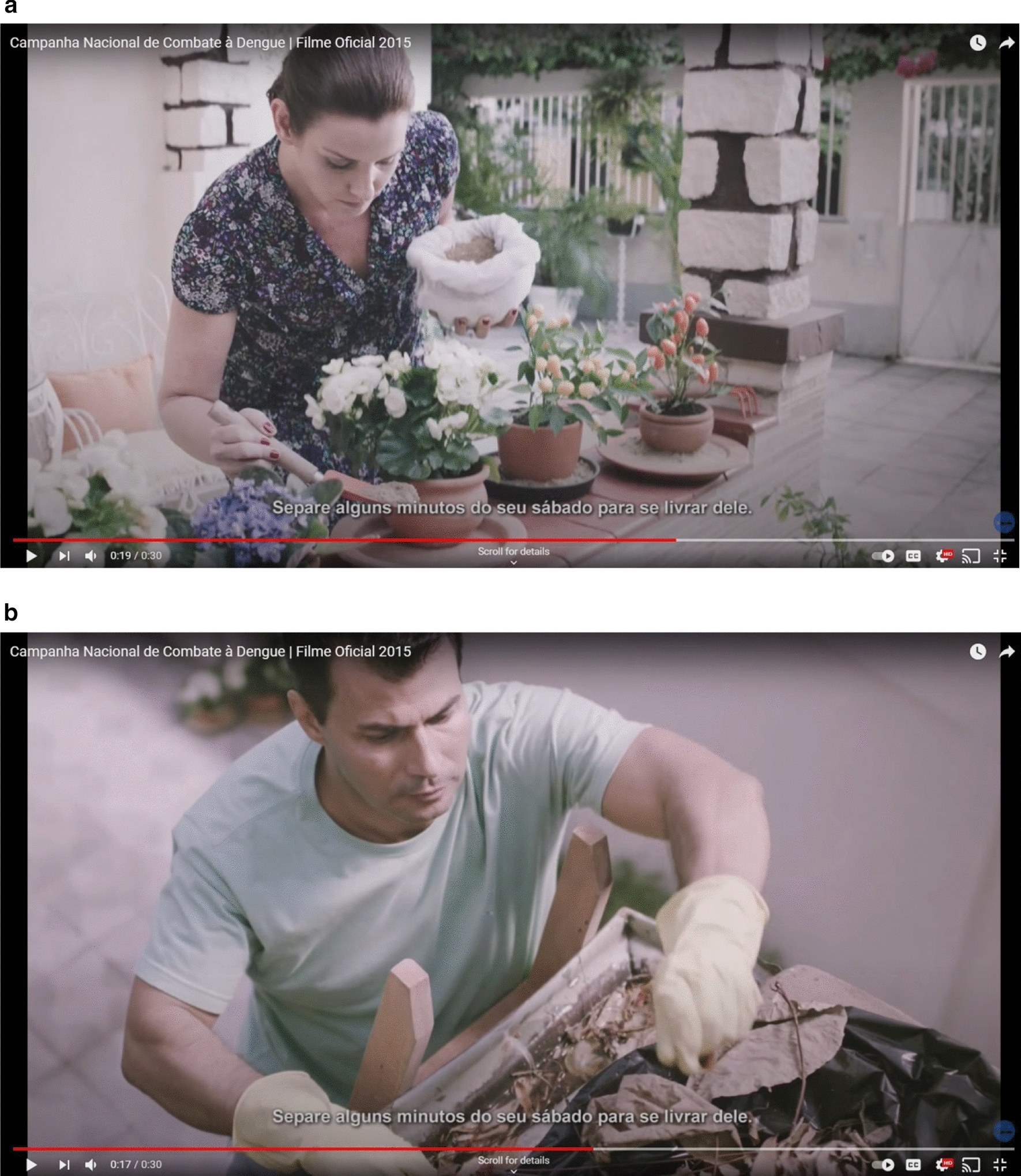
**a** Woman places sand inside the flower vases; **b** A man uses a ladder to reach the roof and clean the gutters

When men were illustrated doing chores, they were performing typically gendered activities, like physically lifting heavy loads. In a widely broadcasted 2015 TV campaign, gender roles were portrayed separately when the narrator suggested the audience should “spend part of their Saturdays doing activities to contain the spread of the mosquito.” While the woman filled the flower vase with sand, her partner was fixing the roof (Fig. 5a, b), portraying gendered stereotypical abilities [5, 49, 50].

Other important aspects of the ads we examine such as pregnancy and contraception management were tacitly communicated. The pamphlet in Fig. 2b includes specific advice directed to females wishing to get pregnant (*Se Deseja Engravidar*) and those who do not (*Se Nao Deseja Engravidar*). In both scenarios, women were advised to visit a health center accompanied by their partners to discuss their options together, with a health professional. The underlying communication message assumed women bore the responsibility of contraception [16, 51], which encompasses informing themselves about the risk Zika imposed on pregnancy, explaining it to their partners and persuading them to discuss Zika prevention with a healthcare professional. Despite the limitations of this piece, it is the only one that portrays contraception management as a couple issue. Most pieces that addressed contraception only presented women.

Additionally, few Zika campaign pieces stated the virus could be transmitted through sex (Fig. 2b). The third paragraph in the 2nd column (pamphlet back) mentioned this possibility and recommended condom use. This content included in the section ‘For those who are Pregnant’ (*Gestante*) neglected women who are not pregnant. The challenges of implementing safe sex, discussed in the next sections, also remained unaddressed.

We now turn to the analysis of focus groups in Recife and Belo Horizonte. Unsurprisingly, women overwhelmingly felt targeted by the Zika campaign.

Participant 1: *That’s all that was discussed* [Zika campaigns focused more on women] *Never it was said: ‘fathers, please, if your wife is…’ That it was never said, in no form of communication. (…) I did not see anything* [any campaign piece] *that talked about it, ‘parents or husbands who intend to have children, be careful not to have Zika, not to transmit to their wives through sex.’ This was never said.*
Participant 2: *The* [campaign] *image, I remember the posters I saw, in institutional environments, even at work, there are two [campaign] images: the mosquito and the woman. You do not see a male figure shown. It's either the mosquito or the woman.* [High SES].
Participant 3: * At least my husband associates Zika with babies. Microcephaly.*
Participant 5: *You would only had heard one thing if you had this session with men. "What there is to talk about Zika? That produces malformations on babies.” Done, it's over. Only that. Men do not have that much interest* [on the topic of Zika]*. They say: "ah, I will not get it; it will not reach me."*
Participant 10: *But then I think it is a matter of information because you hear a lot that it causes microcephaly, so men create a barrier in his mind, that he does not need to protect himself because it affects the baby [not himself].* [Low SES].

Importantly, most women in the focus groups, regardless of social class, criticized this approach, challenging traditional gender arrangements charging women with family healthcare and prevention. Interestingly, some participants tied this communication strategy with the broader public health campaign issue of typically reaching women and not men:

Participant: *I think public health should invest in men. Here in my neighborhood’s health clinic, you see they are having focus groups for pregnant women, diabetics, people with hypertension, adolescents…but if you go in the day, they are having focus groups for adolescents when they have family planning, how many of those adolescents are men?* [Low SES].

This finding summarizes the affirmation that Zika campaigns profoundly relied on heteronormative gender norms.

### What role did gender play in shaping how women navigated Zika and pregnancy prevention during the epidemic with their partners?

“*Women suffer more *[than men]*. Women are born to suffer.*” [Low SES].

We address our second and third research questions by examining the individual level via focus groups. While most women in our focus groups expressed frustration at being targeted by the campaigns, they also expressed essentialist views on why women shouldered the burden associated with family health prevention. That is, the same participants who complained about the focus of the campaigns also elaborated on womanhood intrinsically being tied to care work. For several participants, regardless of SES, motherhood informs female identity even *before* bearing a child, and the fact that women (and not men) *can* become pregnant makes them more aware, interested or responsible for dealing with health-related matters. Participants further reflected on why they thought female characters remained more prevalent in the campaigns than men:

Participant: * Because women live the pregnancy more intensely. Because she is carrying, she has to change her diet, and men do not. (…) I think a mother would feel guiltier if she gets bitten and transmits [Zika] to her baby* [High SES]*.*
Participant 1: * They [men] do not even want to know. The woman is the one who gets worried, same in case of illnesses. You seldom see a man concerned with illness. Is the woman who cares.*
Moderator: * Why?*
Participant 1: * Because the one who gets pregnant is the woman.*
Participant 3: * A woman is more concerned more about her health* [than a man]*. Men do not like going to the doctor.*
Participant 1: * A woman, when she becomes pregnant, she becomes a mother. So, she cares about the baby.*
Participant 3: * When you have a child to raise, you think about yourself. You do your exams regularly. Men do not; if a man goes to the doctor, it's because he's about to die.* [Low SES].

Furthermore, we observed gender norms regarding the care and household labor division were described as ‘cultural’ traits instead of socially constructed differences. These testimonials were more prevalent among high-SES participants than their lower SES counterparts and oftentimes are spoken with a fatalistic tone.

Moderator: * We are going to talk about women and men now. If your* [female] *friends were not using repellent, would their husbands use it (repellent)?*
Participant: * No.*
Moderator: * Why?*
Participant: *Because of this culture* [High SES].
Participant 6: *It is culturally unfortunate that this is still the case; the responsibility is of the mother. If a father abandons his child, nobody judges him or says anything against him, but if a mother abandons her child,* [it is] *everyone, Oh My God, everyone is against her. There is no one who would defend her; it is always like this.* [High SES].

Clearly, c*ulture* was connected to the same female-assigned duties and characteristics—family caring and childcare responsibility. Male partners carrying out their desires despite women’s opposition also emerged subtly through the focus groups. While not prevalent, participants spoke of women discussing an issue in hopes of changing partner behavior. This attempt likely resulted in a conflict, demonstrating women failed in their attempt.

Participant 4: *I think it should be the same* [men and women have the same level of responsibility over contraceptives]*. But in reality, it is not. The woman is the one who takes more attitudes and more responsibility for herself, sometimes (…) Because if she takes the condom to her husband's hand and he does not want to use it, because since she is married, she will give in. That is, she does her part, but he does not cooperate. So folks, ‘we will not be fighting, we won’t keep arguing over a condom.’ We think it is a silly thing, but in reality, it is not.* [Low SES].
Moderator: * And have you two talked about sexual transmission* [of Zika]*?*
Participant 5: * Yes*
Moderator: *And did you start using condoms?*
Participant 5: * No*
Participant 6: * I told my partner: ‘I am with Zika, you will get it.’*
Moderator: *And what happened?*
Participant 6: *He did so much and got it.*

[Parallel talk, laughter].

[Low SES].

All groups asserted a defeatist tone. Yet, the way women navigated this challenge differed by social class.

### Did social class variations significantly affect how women negotiated the Zika infection threat?

‘*Since they do not have that burden on their side, the woman is the one who has to protect herself.*’ [High SES].

*We face everything in silence*. [Low-SES].

Many working-class women expressed how enforcing condom practice with a stable partner had proven challenging. Since men generally disliked condoms, women feared endangering their relationship if they insisted on condom use.

Moderator: *And why does the woman end up giving up?* [having sex without using a condom].
Participant 1: [Because they] *Like the man.*
Participant 2: *To please the partner.*
Participant 3: *Because it's that thing: will he get annoyed and just not want you anymore? So he says: Never mind. He gets angry and does not want you.* [Low SES].

Commonly, low-SES women in our focus groups discussed these difficulties (using the first person) indicating their partners did not like condoms, so they *as a couple* did not use them despite women expressing opposition, in line with previous studies [17–23]. Many females blamed their unintended pregnancies on their partner’s inflexibility.

In contrast, high-SES women often elaborate extensively on their empowerment on this subject. While high-SES women referenced profound gender inequalities across all aspects of Brazilian society, they also described themselves as financially independent, controlling their sexuality and negotiating condom use successfully. Among high-SES women, experiences of low empowerment were articulated subtly, in the third person, referring to friends or family-members’ experiences. High-SES women did not reveal complications associated with contraception management. Nevertheless, some participants acknowledged men did not like condoms, so women had to resort to other methods [63–65]:

Participant 6: [If asked about Zika, some men] *might say, ‘my girlfriend protects herself.’ Done. I have nothing to do with it; she protects herself. Mainly because many men hate to use condoms, it is a very common thing among them* [men]*, the use of condoms, they detest, then compel the woman to use contraceptives, they practically oblige* [women] *because they hate to use condoms. I've seen a lot of this, I have a lot of male friends, and they always say that: ‘I hate using condoms. (…) If she gets pregnant, it's not my fault; she is the one who got pregnant.’* [High SES].

When partner sexual fidelity was brought up, most women expressed that their partners could be unfaithful. Although the result of such negotiations remained unclear, some women even discussed imposing condom use in extra-marital relationships. Often women described those conversations with their partners using light-hearted or playful language.

Participant 1: *So, for me, whatever is fine* [wearing a condom or not]*. I think so…for women, whatever. … as people say here, when they* [men] *use condoms they feel like chewing gum with the plastic wrap, my husband says that. Then I do not know.*
Moderator: *And do you think condoms are bad for women?*
Participant 1: *So, we do not know what partners are doing on the streets, do you understand? They can pick up other women who have diseases and pass them on to people at home. So, with a condom, if they* [men] *accepted, it would be pretty safe. For people at home, for example. For instance, in my case, because I do not trust mine* [my partner]*.*
Moderator: *Got it. Do you trust yours?*
Participant 4: *No*.
Participant 7: *I trust with suspicion.*
Moderator: *So, in connection to the fact that you ‘trust distrusting?’ Do you change your behavior?*
Participant 7: *No* [Low SES].

Consequently, these testimonials suggested men did not openly face conflict with their partners due to refusal to wear condoms. Therefore, the Zika emergency did not threaten masculine privilege.

## Discussion

We set out to examine whether, at the government-level, public health campaigns reinforced heteronormative gender roles; and to investigate, at the individual level, how women navigated sexual health and fertility regulation with their partners in the face of the Zika crisis and the ways in which women’s views and responses to the epidemic varied across social class. As a public health emergency centered on pregnancy, the Zika virus outbreak in Brazil represented an opportunity to expand reproductive health and rights by re-addressing personal responsibilities in Zika prevention, and reducing obstacles to contraceptive implementation.

We provide evidence that Brazilian public health campaigns on Zika heavily focused on mosquito breeding and transmission and relied on conventional gender norms, as in previous campaigns [16, 49–51], reinforcing structural gender inequality [5, 34]. The image of women and the mosquito seem to have strongly helped to increase female vulnerability particularly through downplaying sexual transmission. A pregnant-woman could become infected through her partner, even if she diligently followed all recommendations for preventing Zika. Besides, campaigns failed to engage men, ignoring a significant element in the disease containment. By not widely recommending measures of self protection against mosquito bites to all, campaigns ignored the role of other family members in the transmission, as anyone sharing a household with pregnant women or women at risk for pregnancy should have taken the same personal protection measures against mosquito bites.

The content analysis also shows that communicating Zika fetal development risks overwhelmingly embraced pregnancy and measures of personal care, targeting women with the use of female characters and pronouns, pastel colors, and gendered messages appealing to the female role as family health custodians. Campaigns disproportionally emphasized pregnant women as the population at risk, overlooking the fact that non-pregnant reproductive-aged women who were sexually active would not feel susceptible to the risk of Zika and consequently did not adapt their behavior.

Zika health campaigns failed to provide a ‘toolkit’ for handling contraceptive management and sexual interactions within stable unions. The very few pieces that addressed contraception management expected women to assume the prevention burden, fomenting uneven responsibilities distribution [34]. Ads relied on the perplexing assumption that women would be able to implement their fertility decisions, despite ample information on the country’s high rates of unintended pregnancy, as well as the sparse condom use reported by low-SES women [45].

Very few campaigns mention sexual transmission and condom use, and none of them targeted at the entire population of sexually active people; none of them were a short piece. Focus groups data shows how gender power differentials influenced women’s ability to negotiate condom use, critical to female sexual protection [17, 23]. As a result, Zika campaigns did not challenge gendered-based power dynamics governing sexual protection and contraception in Brazil and did not contribute to expand knowledge on women’s reproductive rights and women’s health services available free of charge, neither did they initiate discussion regarding gender equity policies needed during the outbreak as previous gender scholars have pointed [5, 30, 32, 33].

In line with the results of the content analysis, our focus group findings suggest that overwhelmingly, and across social classes, women consistently claimed men held little interested in the disease, associating Zika with pregnancy. In this vein, women assumed their responsibility for Zika prevention as extending their customary roles as “contraceptive experts” and the primary party responsible for home cleanliness and protecting their bodies, as motherhood was women's natural proclivity. Research for the United States shows that prevalent cultural perception purporting men were not committed to pregnancy prevention explained why women usually were charged with this duty, legitimizing traditionally gendered roles in order to not compromise health [63]. As Campo-Engelstein [64] also demonstrated, dominant masculine ideologies have inhibited [65] female trust in males engaging with sexual and reproductive health. These biological, essentialist perceptions, legitimized traditionally gendered labor in which women were responsible for preventing the Zika virus from spreading because those responsibilities inevitably fell into the female realm. In turn, failed attempts to alter sexual behavior, inability to disagree with their sexual partners and women’s own perception of their role as contraceptive experts comprised vital aspects for understanding female sexual behavior.

Our focus group analysis suggests that both low- and high-SES women were discontent with male disengagement with parenthood, contraception, and healthcare. A number of women contended their partners refused their requests to wear condoms, arguing they interfered with sexual pleasure; male partners mentioned no need for condoms since they were faithful and monogamy eliminated the precautionary measure. Yet, the way power unbalances appeared varied across social class. Some low-SES women feared to destabilize their relationship or lose their partners if they insisted on condom use. Frequently, those low SES women admitted conceding to their partners despite knowing it would likely result in disease transmission or unplanned pregnancy. Many low-SES women report only gaining contraceptive knowledge and empowerment after having experienced their first pregnancy, usually unplanned. Therefore, the Zika battle missed an opportunity to improve women’s status, increase egalitarian care division and reduce female vulnerability by engaging men in public health efforts related to sexual and reproductive outcomes.

High-status women, on the other hand, frequently described themselves as empowered, enforcing condom use when confronted with male refusal. These participants also took the pill or resorted to another contraception form with high efficacy, which require more planning and resources, but what helps explain their lower levels of unplanned pregnancy. Although further study of Brazil is needed, testimonies of these women shed further light on the complexity of low-SES sexual and fertility decisions.

Nevertheless, the same high-status women who acknowledged gendered-power unbalance explained the uneven distribution of care work in their households and expressed discontent with these “cultural” arrangements. When high-SES women articulated the word “culture” to explain male privilege, they described an inherent order that perpetuated gender inequality among the upper class, but that they do not fight. So, although higher educational attainment protects women’s bargaining power and sexual health significantly [66, 67], the socioeconomic advantage did not completely prevent high-SES women from the conventional gender norms during partner interaction. Scholars exploring gendered patterns in housework allocation exemplified while double-earner, high-SES couples shared more household responsibilities, women still performed more tasks [58, 68].

It is important to point out that we also found exceptions to the noted patterns. Some women, from high and low SES, in our focus groups, did declare their partners actively participated in contraceptive habits and household tasks, remaining concerned about the Zika epidemics. These examples led us to believe the Brazilian communicational campaigns also missed the opportunity to support and promote non-conformative gender representations of masculinity among men.

This study presents several avenues for future research. Contrasting with the progressive renaissance prevalent in the 1980s when the anti-HIV strategy was implemented, Zika emerged during a conservative resurgence in Brazil. Strongly conservative evangelical groups recently gaining hold in Brazilian politics generally oppose sexual and reproductive rights [69]. There are reports of abortion pills confiscation [27], and requests to increasing sentence for women who get abortions and anyone who performs abortion of a fetus with microcephaly [70]. The possible impact of this political shift on public health communication strategies should be investigated more in-depth as Zika campaigns promoting condom use likely conflict with conservative values, consequently, unleashing political backlash.

A second and necessary avenue for future study is to study the possible long term impacts of the Zika epidemics in the life course. Our focus groups were conducted in 2016. It is possible that the Zika epidemics has created important changes in sexual behavior, in gender relations and in reproductive target reconsideration.

Lastly, focus groups might compromise obtaining sensitive information. Yet, Brazilian women discussed extensively sensitive issues such as their partner infidelity and male contraceptive habit disengagement, leading us to conclude the group organization seemed to have fostered female participant willingness to share personal experiences. Future studies should address male’s perception of campaigns and of their own role in reproductive behavior.

## Conclusion

This paper explored how gender conventions shaped government communication campaigns during the Zika epidemic and how women navigated this public health emergency tapping directly into gender-based power dynamics within sexual relationships. Our findings suggest gender constituted a persistent system of social practices acting in multiple relational contexts in the Zika epidemics: individually (micro) through interpersonal relationships and governmental-level (macro) through communication campaigns. We demonstrated that both levels sustained stereotypical male and female beliefs fomenting female vulnerability to Zika virus infection.

Zika and microcephaly campaigns focused too much on mosquito transmission and relied entirely on motherhood concepts that created different expectations for men and women. We showed how health officials drew on a traditionally-gendered *script* largely situating pregnant women as responsible for averting mosquito bites and microcephaly and by epitomizing Zika as a ‘pregnant woman’s issue,’ campaigns ignored the large number of unplanned pregnancies in Brazil and devalued the contributions that other members in the household could be for the containment of the disease.

We also found that many Brazilian women confronted Zika emergency sexual health and fertility decisions from a disadvantaged position with respect to their partners. Thus, there was a disconnection between recommending measures of personal care and pregnancy postponement—even if informally—and not addressing the issues which interfere with a women capability to fully exercise their reproductive rights. Since low-socioeconomic status (SES) women possessed fewer resources to preclude infection, we also find that beyond the gender divide, low SES women faced more pronounced Zika prevention challenges as destroying mosquito breeding sites, implementing measures of individual protection and preventing pregnancy is nonetheless more troublesome for those in the low end of the socioeconomic scale [5]. By not taking into account the large socioeconomic and self-determination differences of Brazilian women, campaigns not only charged women with the responsibility to avert microcephaly [5, 34], but also “transforms a problem that is political, systemic, and structural into a question of individual conduct of poor, marginalized women who do not have power over their life projects” [71].

In addition to continuing alerting about the necessity of destroying mosquito breeding sites and getting rid of standing water, future campaigns should include the following elements: Recommendation of measures of self-protection against mosquito bites to all people, especially sexually active and people who share a household with a pregnant woman or sexually active woman; Raise awareness about sexual transmission of Zika; Inform non-pregnant women about high rates of unplanned pregnancies; Inform population about sexual and reproductive health services available free of charge in public clinics and inform the benefits of using long active reversible contraception combined with condom use; Raise awareness of sexually active men on their primary role in preventing Zika transmission through protected sex and protection against mosquito bites; Raise awareness of sexually active men on their shared responsibility on pregnancy prevention; Promote non-conformative gender representations of masculinity.

We recognize that Brazilian policymakers and health managers, given their long tradition of public health campaigns, had the best intentions when they opted to reproduce Dengue strategies and focus on women and their babies, the most vulnerable population in regards to Zika. But making recommendations without disrupting the existing conditions of structural gender inequality increased female’s vulnerability. Especially in contexts of health emergencies, it is mandatory to anticipate constraints on women's right to exercise their sexual and reproductive rights [16]. These lessons are important when dealing with all public health crises, including the Covid-19 pandemic.

### Supplementary Information

**Additional file 1.** Focus groups protocol. Contains protocol utilized in all focus groups (translated to English).

**Additional file 2. **Additional Table. Table contains description of print Zika public health dissemination campaign materials by main messages and lengths of print material. Brazil, May 2016–May 2017. *N* = 72. Percentages in parenthesis.

**Additional file 3.** Additional Figures. Contains additional examples of Zika TV campaigns portraying gender stereotypes.

## Data Availability

All public campaign materials are publicly available. The dataset analyzed during the current study are available from the corresponding author on reasonable request. Focus groups transcripts are not publicly available due research ethics protocol agreement, but are available from the corresponding author on reasonable request.

## Abbreviations

WHO: World Health Organization
SES: Socioeconomic status
HIV: Human immunodeficiency virus
AIDS: Acquired immunodeficiency syndrome
SRH: Sexual and reproductive health
STI: Sexually transmitted infection

## Footnotes

1 Visiting the websites on January 2021, we noticed that new material had been included that was not available for download during the period of campaign pieces collection; as a result, it was not included in the analysis

2 Unfortunately, we are unable to track how many of the pieces produced by the federal government were reproduced by the local agencies and in which frequency.
