# Correction: Zika virus public health crisis and the perpetuation of gender inequality in Brazil

**DOI:** 10.1186/s12978-024-01846-6

**Published:** 2024-08-19

**Authors:** Raquel Zanatta Coutinho, Aida Villanueva, Abigail Weitzman, Letícia Junqueira Marteleto

**Affiliations:** 1 Faculdade de Ciências Econômicas, Universidade Federal de Minas Gerais and Center for Development and Regional Planning (Cedeplar), Av. Pres. Antônio Carlos, 6627-Pampulha, Belo Horizonte, MG 31270-901 Brazil; 2 Department of Sociology, University of Massachusetts-Amherst, 910 Thompson Hall, Amherst, MA 01003 USA; 3 The College of Liberal Arts, University of Texas at Austin and Population Research Center, 116 Inner Campus Dr Stop G6000, Austin, TX 78712 USA

**Correction: Reprod Health 18, 40 (2021)**

**https://doi.org/10.1186/s12978-021-01067-1**

Following publication of the original article [1], the authors reported that the second author's name should be “Aida Villanueva” instead of “Aida Villanueva Montalvo”.

The correct author’s name has been provided in this Correction.

The original article [1] has been corrected.
