# Supplementary material for: Zika virus public health crisis and the perpetuation of gender inequality in Brazil
Source: Reprod Health. 2021 Feb 15;18:40. doi: 10.1186/s12978-021-01067-1 (PMC7883759; doi:10.1186/s12978-021-01067-1)
Supplement: Supplementary file 1 — Additional file 1. Focus groups protocol. Contains protocol utilized in all focus groups (translated to English). [file 12978_2021_1067_MOESM1_ESM.docx]

# ADDITIONAL FILE 1

# Methodological Appendix

**Healthcare Demand and Supply During the Brazilian Zika Epidemic:**

**Focus Group Protocol**

Greet participants as they arrive.

Distribute Consent Form and Number tag.

Walk participants to their seat.

***Turn on audio recorder****

*Before questions begin*: Read and confirm informed consent out loud. Assistant collects signed content forms.

Thank you so much for your participation. Let’s begin.

*Moderator introduces herself.*

1. Do you have children, or have you thought about having children?

2. Would you like to have (more) children in the future?

3. What have you heard about the Zika virus? Where did you hear this information? What did you hear?

4. Do you worry that you or your family will become infected with the Zika virus? What do you think you would do if someone became infected?

5. Do you know anyone who has been infected with the Zika virus? Who? What do you know about the case?

7. Has the Zika epidemic changed the behaviour of anyone you know or heard of thinking in day-to-day life? If so, how?

8. Have you been to a health clinic or hospital since the start of the epidemic (for any reason)? If so, how was your visit? Did the doctor or nurse discuss the Zika virus with you?

9. Has the epidemic changed the way you or someone you know think about having children?

10. Is anyone you know currently trying to avoid pregnancy? How?

11. Do you think it has become easier, harder, or the same for women to prevent pregnancy right now compared to a year ago? Why?

12. Has the Zika virus changed your husband or boyfriends’ attitudes toward contraception and sex? Has it changed for any men you know?

13. If your sister or friend was pregnant and became infected with the Zika virus, what would you think? How would you react?

*To end: Thank the group again for their participation.* *Offer your contact information (which was also available on the recruitment flyer and Informed Consent) and remind participants that they can follow up with you if they have questions at any time.*

*** Turn off audio recorder. ***
