# Supplementary material for: Zika virus public health crisis and the perpetuation of gender inequality in Brazil
Source: Reprod Health. 2021 Feb 15;18:40. doi: 10.1186/s12978-021-01067-1 (PMC7883759; doi:10.1186/s12978-021-01067-1)
Supplement: Supplementary file 2 — Additional file 2. Additional Table. Table contains description of print Zika public health dissemination campaign materials by main messages and lengths of print material. Brazil, May 2016–May 2017. N = 72. Percentages in parenthesis. [file 12978_2021_1067_MOESM2_ESM.docx]

# ADDITIONAL FILE 2

**Table 1: Description of print Zika public health dissemination campaign materials by main messages and lengths of print material. Brazil, May 2016-August 2017. N= 72. Percentages in parenthesis.**

|  |  | **Lengh of text** | |
| --- | --- | --- | --- |
|  |  | **Short** | **Long*** |
|  |  | **51** | **21** |
| Eliminate Standing Water | No | 14 | 1 |
|  |  | (27.45) | (4.76) |
|  | Yes | 37 | 20 |
|  |  | (72.55 | (95.24) |
| Personal protection against bites | No | 48 | 11 |
|  |  | (94.12) | (52.38) |
|  | Yes | 3 | 10 |
|  |  | (5.88) | (47.62) |
| Special care during pregnancy | No | 47 | 13 |
|  |  | (92.16) | (61.90) |
|  | Yes | 4 | 8 |
|  |  | (7.84) | (38.10) |
| Information about Zika Symptoms | No | 46 | 7 |
|  |  | (90.20) | (33.33) |
|  | Yes | 5 | 14 |
|  |  | (9.80) | (66.67) |
| Seek health care | No | 45 | 12 |
|  |  | (88.24) | (57.14) |
|  | Yes | 6 | 9 |
|  |  | (11.76) | (42.86) |
| Pregnancy counseling | No | 48 | 18 |
|  |  | (94.12) | (85.71) |
|  | Yes | 3 | 3 |
|  |  | (5.88) | (14.29) |
| Condom use or contraception | No | 51 | 18 |
|  |  | (100.00) | (85.71) |
|  | Yes | 0 | 3 |
|  |  | (0.00) | (14.29) |
| Microcephaly | No | 43 | 16 |
|  |  | (84.31) | (76.19) |
|  | Yes | 8 | 5 |
|  |  | (15.69) | (23.81) |
| Female Figure | No | 44 | 14 |
|  |  | (86.27) | (66.67) |
|  | Yes | 7 | 7 |
|  |  | (13.73) | (33.33) |
| Male Figure | No | 46 | 17 |
|  |  | (90.20) | (80.95) |
|  | Yes | 5 | 4 |
|  |  | (9.80) | (19.05) |
| Main perceived audience | General public | 31 | 6 |
|  |  | (60.78) | (28.57) |
|  | Person responsible for household | 15 | 9 |
|  |  | (29.41) | (42.86) |
|  | Women | 5 | 6 |
|  |  | (9.80) | (28.57) |

Source: Communication campaigns pieces downloaded directly from public resources at each institutional website (Ministério da Saúde, MG State, PE State, Townhouses of Belo Horizonte and Recife)

Notes: Long text materials comprised of Brochure, Folder and Infographic
