# Supplementary material for: Zika virus public health crisis and the perpetuation of gender inequality in Brazil
Source: Reprod Health. 2021 Feb 15;18:40. doi: 10.1186/s12978-021-01067-1 (PMC7883759; doi:10.1186/s12978-021-01067-1)
Supplement: Supplementary file 3 — Additional file 3. Additional Figures. Contains additional examples of Zika TV campaigns portraying gender stereotypes. [file 12978_2021_1067_MOESM3_ESM.docx]

# ADDITIONAL FILE 3

**ADDITIONAL FIGURES**


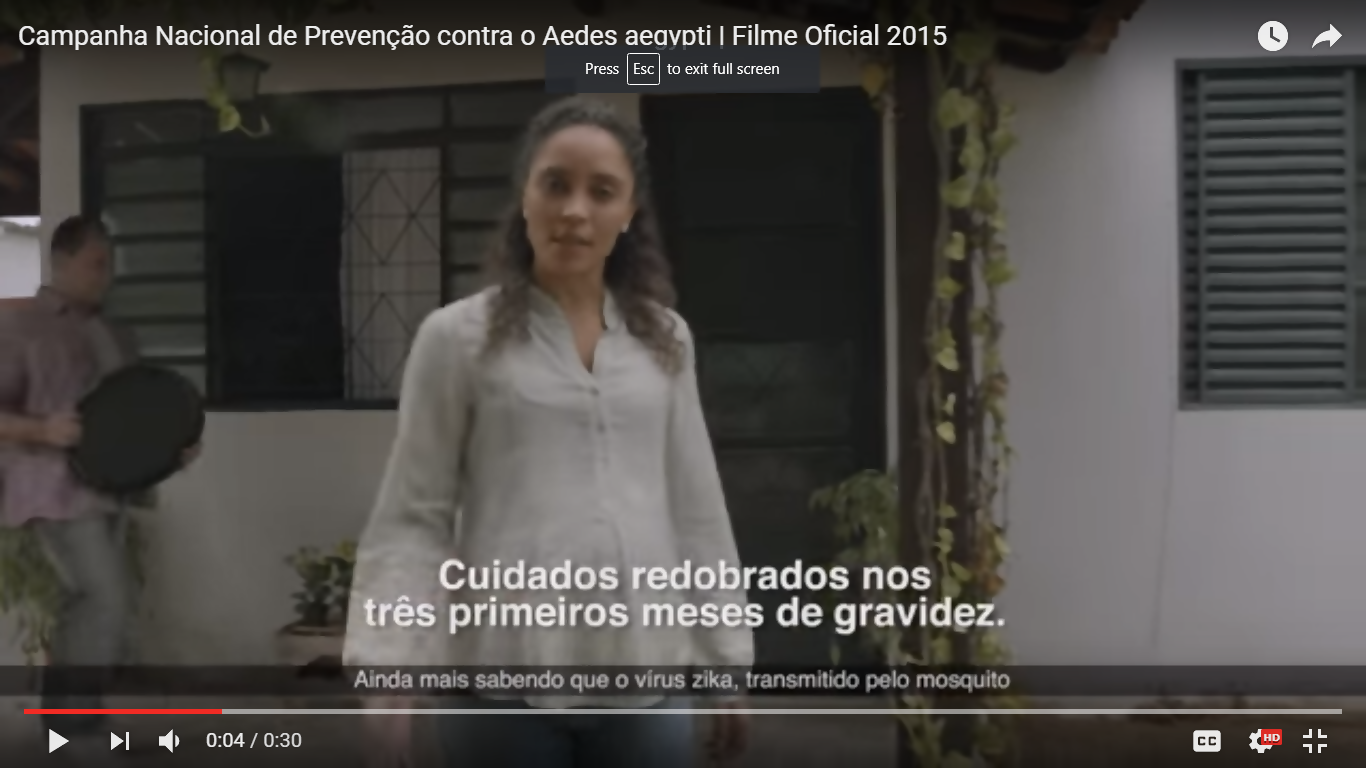


Figure 1a: a pregnant woman gives advice about being careful during pregnancy.


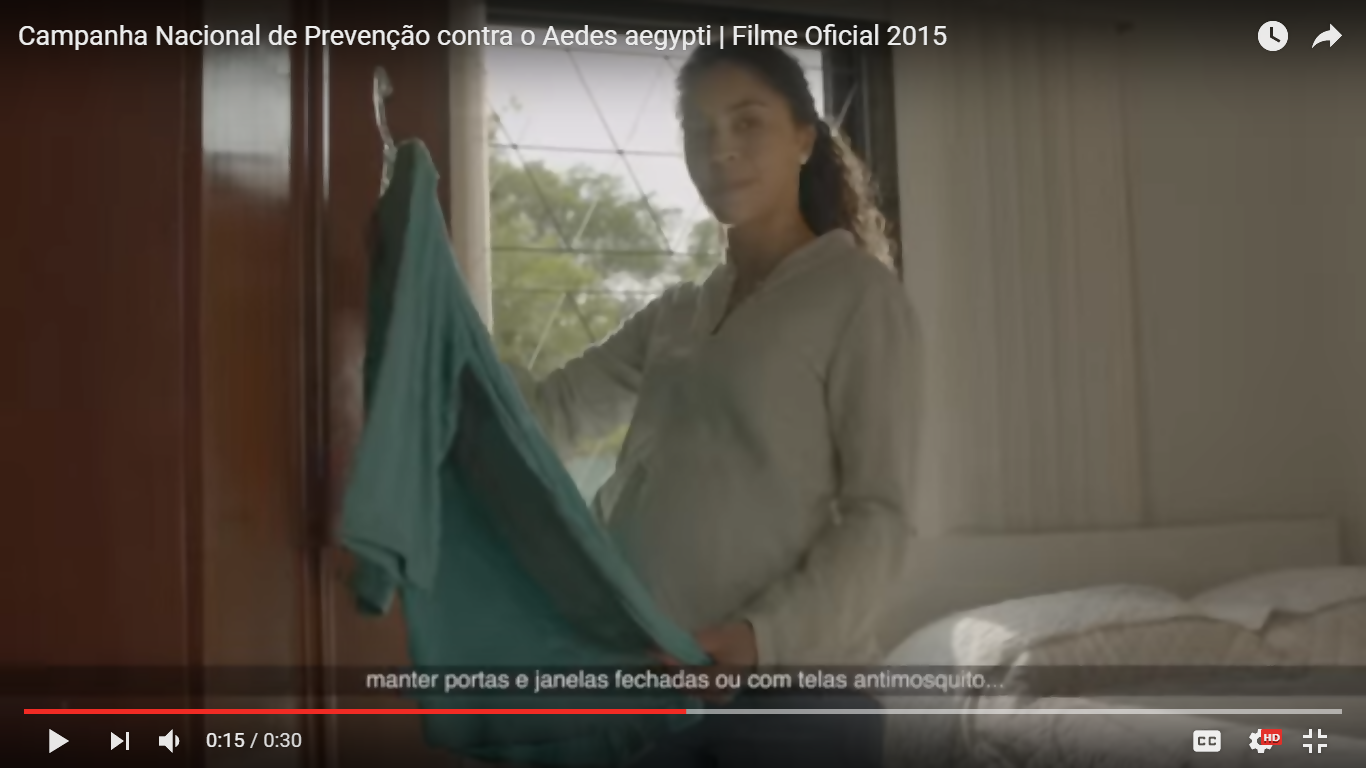


Figure 1b: a pregnant woman talks about the importance of wearing long sleeve clothing.


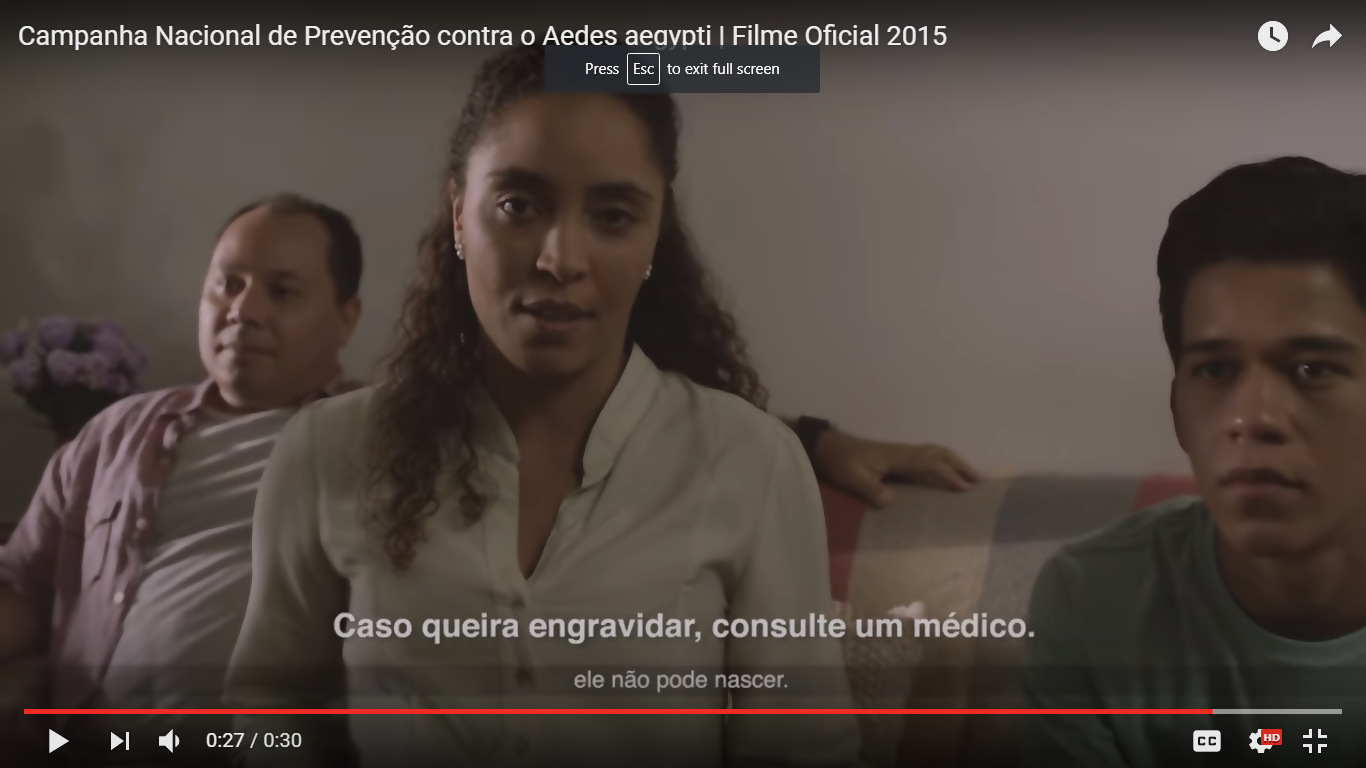


Figure 1c: a pregnant woman tells female audience to protect themselves while her supposedly husband and adolescent son watch TV.
